# BioPAN: a web-based tool to explore mammalian lipidome metabolic pathways on LIPID MAPS

**DOI:** 10.12688/f1000research.28022.1

**Published:** 2021-01-06

**Authors:** Caroline Gaud, Bebiana C. Sousa, An Nguyen, Maria Fedorova, Zhixu Ni, Valerie B. O’Donnell, Michael J.O. Wakelam, Simon Andrews, Andrea F. Lopez-Clavijo

**Affiliations:** 1 Bioinformatics Group, Babraham Institute, Babraham Research Campus, Cambridge, CB22 3AT, UK; 2 Lipidomics facility, Babraham Institute, Babraham Research Campus, Cambridge, CB22 3AT, UK; 3 Institute of Bioanalytical Chemistry, Faculty of Chemistry and Mineralogy, Center for Biotechnology and Biomedicine, Universität Leipzig, Leipzig, 04109, Germany; 4 Systems Immunity Research Institute, School of Medicine, Cardiff University, Cardiff, CF14 4XN, UK

**Keywords:** LIPID MAPS, Lipidomics, Biosynthetic pathway analysis, lipids, lipid profiling.

## Abstract

Lipidomics increasingly describes the quantitation using mass spectrometry of all lipids present in a biological sample.  As the power of lipidomics protocols increase, thousands of lipid molecular species from multiple categories can now be profiled in a single experiment.  Observed changes due to biological differences often encompass large numbers of structurally-related lipids, with these being regulated by enzymes from well-known metabolic pathways.  As lipidomics datasets increase in complexity, the interpretation of their results becomes more challenging.  BioPAN addresses this by enabling the researcher to visualise quantitative lipidomics data in the context of known biosynthetic pathways.  BioPAN provides a list of genes, which could be involved in the activation or suppression of enzymes catalysing lipid metabolism in mammalian tissues.

## Introduction

Lipids (fats) are essential and diverse families of molecules that play structural, energy storage and signalling roles. They are connected through complex metabolic pathways, which comprise linked series of enzymatic reactions (several are outside cells, *e.g.* PLA2 isoforms, autotaxin). Thus, lipids can be substrates, products or intermediates. It has been estimated that there are approximately 3–5000 different lipid species in mammalian cells although the true number is still unknown and extremely difficult to reliably measure ^1, 2^. In recent years, great advances have been made in our ability to experimentally determine the elemental composition and quantitation of lipid levels in biological samples, with the advent of rapid scanning benchtop mass spectrometers (MS), in particular high-resolution configurations such as Orbitrap, and ToF. Liquid chromatography-MS, either tandem or high resolving power, interfaced with processing pipelines such as Lipid Data Analyser (LDA), LipidFinder, LipidHunter, MS-Dial, XCMS and many others makes it feasible to simultaneously monitor the dynamic changes in hundreds of lipid molecular species in biological samples ^3– 7^.

As the quantity and detail of quantitative lipid data continue to grow it has become considerably more challenging to interpret the complex sets of changes within the lipidome ^8^. Relevant biological perturbations usually happen not at the level of a single lipid molecular species in isolation, but as broad sets of changes over entire lipid classes and subclasses ^9^. For example, a group of phosphatidylcholines will tend to change together in the same direction, since they are being regulated by the same enzyme isoform(s). This introduces characteristic patterns in the data that can reveal important clues as to the underlying level of genetic and transcriptomic regulation. Consequently, there is a need for software tools to automate and facilitate biosynthetic pathway scale analyses with lipids grouped according to structural motifs. Nguyen *et al.* and Hann *et al.* described the analysis of quantitative lipidomics datasets at pathway levels ^10– 12^. Here, we have integrated the biosynthetic metabolic levels into BioPAN. So, BioPAN combines current knowledge of lipid metabolism with a statistical analysis functionality, by comparing two biological conditions to identify activated or suppressed pathways, and present the results in an interactive graphical display. BioPAN works with data resolved to the level of individual lipid molecular species, but it can aggregate results at the subclass level to simplify the interpretation. Mammalian reaction information is converted into a metabolic pathway within BioPAN, allowing it to predict the most likely lipid transforming genes modulated by an experiment, to provide direct biological insight and generate hypotheses that can be experimentally tested.

BioPAN is openly available on the LIPID MAPS ^®^ Lipidomics Gateway, at https://lipidmaps.org/biopan/. The tool is designed to allow users to upload and analyse their own data, and example datasets are also provided for evaluation. BioPAN is fully integrated with LipidLynxX, allowing users to convert different naming (short notation) conventions from diverse software inputs to be able to make it readable on BioPAN. Additionally, LipidLynxX will convert lipid results with fatty acid position, double bond location, and stereochemistry into a sum composition of carbon number and double bond equivalents. For example, DAG (16:0/20:1(Z11)), is converted by LipidLynxX to DG 36:1. BioPAN offers a link with LIPID MAPS Structure Database (LMSD) ^9^.

## Methods

### Implementation

The statistical model used in BioPAN was originally described by Nguyen *et al.* ^10^. In brief, BioPAN uses data from replicated quantitative lipidomics experiments with two biological experiments: a condition of interest (treated) and a control condition. BioPAN calculates statistical scores for all possible lipid pathways to predict which are active or suppressed in the treated samples compared to the control set.

The BioPAN workflow relies on the calculation of the Z-score, which considers the mean and the standard deviation of the experiment assuming a normally distributed data of lipid subclasses. A probability function (P) is computed and subtracting from one to obtain Q, which in turn is the probability that a Z-score is due to chance. The Z-score is then used to predict whether a particular reaction is significantly ( *p* < 0.05) changing between control and treated conditions. Z-scores for all reactions in a pathway are combined using a cumulative function (CDF) to give a global pathway Z-score The set of equations used to calculate Z-scores are presented and discussed elsewhere ^10^. Changing reactions are classified as activated or suppressed depending upon the direction of change. BioPAN, by default, calls a reaction or pathway as significantly modified at a level of *p* < 0.05 (Z > 1.645).

### Operation

BioPAN is implemented as an open access web-based tool. The front end uses HTML, jQuery (v3.5.1) for the interface and the Cytoscape.js library (v3.10.2) ^13^ to draw and manipulate the results graphs. The server-side code uses PHP (v7.4.11) for request handling and R (v4.0.2) for data processing and statistical analysis. BioPAN runs on all modern web browsers and is available at https://lipidmaps.org/biopan/.

BioPAN allows uploading of lipidomics datasets containing quantitative data from two different experiments or conditions ( *e.g.* wild type against treated/knock-out). The use of BioPAN is simple and intuitive with a user-friendly interface. The data files should have minimum two replicas per condition, as BioPAN compares the changes between the experiments. BioPAN takes an input file in .csv (Comma-Separated Values). The first column on the file should include the lipid abbreviation subclass followed by the notation *e.g.* PC 38:4, PC 18:0_20:4, PC 18:0/20:4, or PC 18:0/20:4(5Z,8Z,11Z,14Z), for glycerophopholipids, glycerolipids, and sterol lipids. Sphingolipid molecular species written as *e.g.* d18:1/20:4, or 18:1;O2/20:4, where 18:1;O2 is the sphingosine base and 20:4 is the N-acyl chain liked to the sphingosine base are also recognised in BioPAN. Non-conventional nomenclature like DG(aa-34:0, DG(ea-34:1) is not recognised by BioPAN and the user manually need to change the lipid subclass abbreviation ^14^. There are additional instructions about the structure of the data file following the link https://lipidmaps.org/biopan/doc/step1.html, which directs the user to each lipid subclass abbreviation in LIPID MAPS ^®^ Lipidomics Gateway.

The first step in BioPAN is to load a file of quantitative lipidomics data. After uploading, BioPAN uses the LipidLynxX ^15^ tool to cross-match some lipid names into the LIPID MAPS ^®^ Lipidomics Gateway nomenclature style according to the guidelines from COMP_DB ( https://www.lipidmaps.org/resources/tools/bulk_structure_searches_documentation.php) ^9, 14, 16^. BioPAN then classifies the submitted lipids as either unrecognised, processed, or unprocessed. Unrecognised molecular species are lipids whose subclasses are not included into BioPAN database, while unprocessed species are part of BioPAN, but were not associated with any reactions. BioPAN’s database was manually collated followed by a validation using available literature. The database currently contains 94 lipid reactions identified in mammals, covering 41 lipid subclasses. Only processed molecular species, *e.g.* those which can be associated with at least one reaction, are used for downstream analysis.

**Figure 1.**
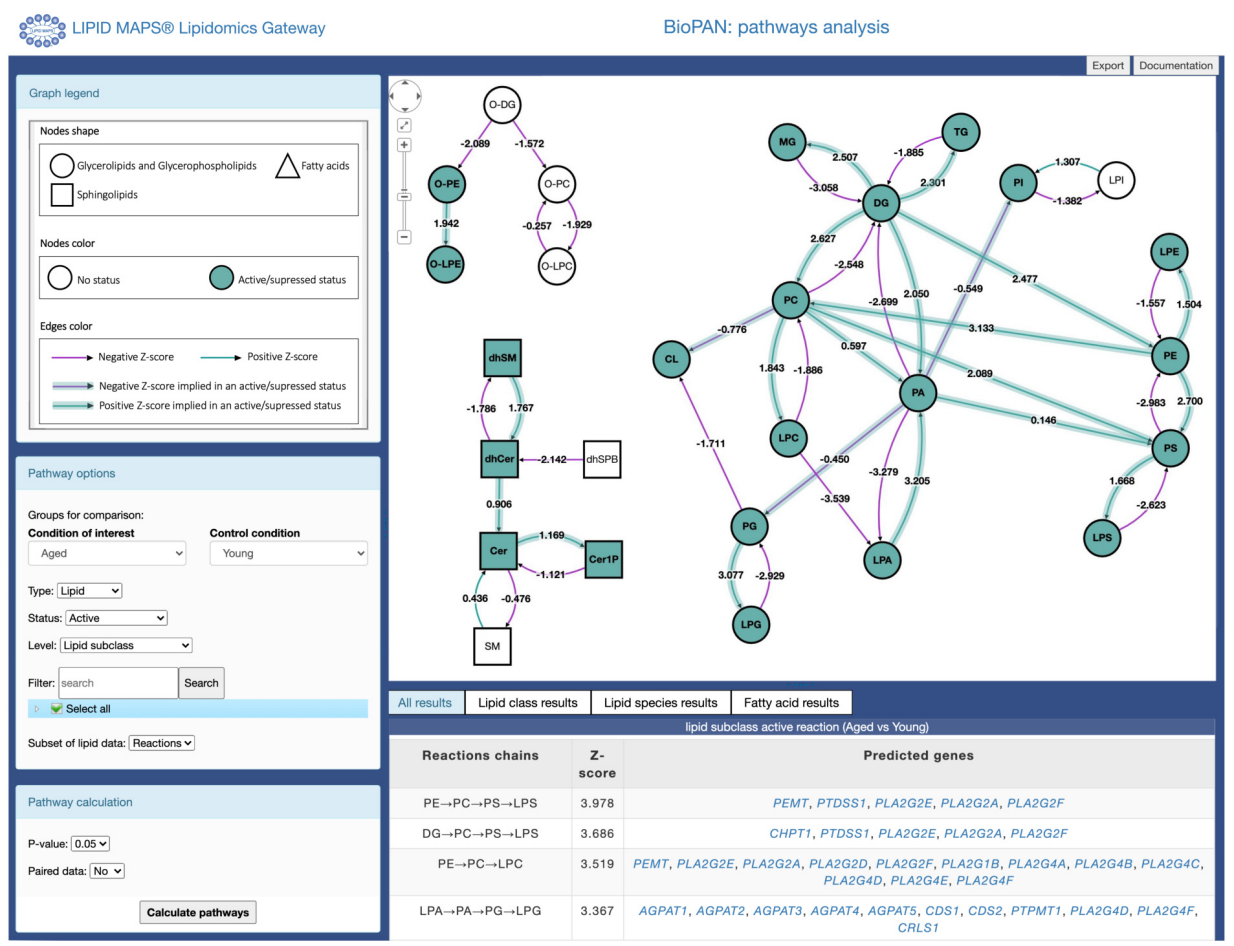
Main results page view for BioPAN. Pathways are displayed as an interactive graph at the centre of the screen. Users have control over the information presented in the graph using the menus on the left side panel and results tables are displayed at the bottom of the screen.

The second step within BioPAN requires users to associate each sample with a condition. ( *e.g.* sample names: control1, treated1, or control2, treated2, and so on, with their respective replicates). Each condition requires a minimum of two replicas (three or more replicas per condition are strongly recommended). Once assignments have been made, BioPAN searches for reactions and pathway changes between these. Following analysis completion, the user is directed to the results page ( Figure 1). The main viewport shows an interactive graph comprising lipids and reactions assigned to the lipids included in the input file uploaded. The user can select how the pathways are displayed in the interactive graph by manually moving the nodes in the graph, while the link between product and substrate is maintained. Thus, providing a view of all possible reaction pathways. The side panel provides a legend for the graph and controls to modify the specifics of the analysis performed and the level of detail in the graph. The legend describes the shape of the nodes, which denote the lipid subclass, and the colour of the nodes represent the pathway status (active/suppressed or none). Each arrow between two nodes (lipid subclass/lipid molecular species) depicts the reaction direction between the two lipids. The colour of the arrows depends on the value of the Z-score (see the *Implementation* section), where green indicates a positive Z-score and purple negative, as shown in Figure 1. The user can click in the arrows connecting each node and a pop table is generated by BioPAN showing all the lipid molecular species selected and non-selected in the reaction between two nodes (see Extended Data, Table S-1). Selecting the arrow is only functional when selecting the lipid subclass level in the pathway options (described in the section below). Additionally, information on lipid subclasses and molecular species are available by clicking on the nodes, linking to the LMSD database ( https://www.lipidmaps.org/data/structure/index.php).

The bottom panel shows a tabular view of statistically significant ( *p* < 0.05 equivalent to Z-score > 1.645) results.

***Display options.*** The pathway options on the left of the screen allow users to customize the display of results in the graph. BioPAN arbitrarily chooses two conditions to compare (condition of interest and control condition, see Figure 1. The user can change conditions to compare using the drop-down menu ( Figure 1). Users can choose to view pathways based on either the lipid subclass or on the fatty acyl composition. They can also choose which pathways to highlight in the main graph. Users can opt for either significantly active (condition of interest > control) or significantly suppressed (condition of interest < control) pathways. Where the same reaction can participate in multiple pathways, users can also choose to view only the most active (or most suppressed) pathway. This will highlight only the pathway with the highest individual pathway level Z-score for pathways containing reactions where a substrate is the starting agent in a reaction. In other words, selecting most active or most suppressed *status* still follow the rule for the active/suppressed status in terms of colours and Z-scores as discussed above. However, the Z-scores in an active/suppressed single reaction ( *e.g.* PC→DG) are compared for substrates, intermediates, and products following each step of the pathway. For example, in the cerebral cortex (with pathway options: active status, subclass level, reaction subset of lipid data, 0.05 *p* value, no paired-data), reactions within multiple pathways like PC→DG→MG and PC→DG→PA. PC is the initial substrate and have individual reaction Z-scores of 3.083 (PC→DG), 2.054 (DG→MG), and -0.408 (DG→PA). It can be observed above that PC→DG is common for the first step in both pathways, and then BioPAN selects the most active reaction with the highest Z-score for the second part of the pathway. So, the Z-score of DG→MG is greater than the score for DG→PA. Thus, PC→DG→MG is most active pathway compared to PC→DG→PA. There might be more than one active pathway with one lipid-initiated substrate ( *e.g.* DG→TG, DG→PE→PS, and DG→PC→CL), the same principle is applied, and BioPAN will consider only one of those pathways as most active.

Users can also choose to have the nodes in the graph aggregated into lipid subclasses, or they can view individual lipid molecular species. All views allow for filtering to display only a user-selected subset of lipids. These can be chosen either from a tree of checkboxes for each subclass, or using a free text search, allowing users to focus on particular lipids of interest (see Extended Data, section S-2, Figure S-1). Alternatively, the user can opt to view only reaction chains which occur within the context of a set of curated named pathways stored within BioPAN ( *e.g.* biosynthesis of PC and Kennedy pathway). Changing pathway options affects both the graph and results tables.

***Calculation options.*** Under the Pathway Calculation section, the user can control details of the statistical calculation. They can alter the groups to be compared as well as changing the threshold for significance from the default of *p* < 0.05. If replicates in the uploaded data were generated as matched pairs ( *e.g.* control and treated sample coming from the same animal) then the calculation can also take this into account in the *t*-test step of the analysis.

***Exporting results.*** Results of the statistical analysis of active/suppressed pathways are presented in four tables at the bottom of the page, where predicted gene changes can be visualized. Export options are also available on the top right corner in the BioPAN viewport, where the main graph can be exported in several formats (JPEG / PNG / JSON / TXT) and tables are exported as TSV files.

## Example use case

Lipidomics data from cerebral cortex and liver of young and aged mice ^17^ were used here as an example to illustrate how to use BioPAN and interpret its results. Ando *et al*. ^17^ compared the lipid profile of different tissues in young and aged mice with the aim of detecting age-related as well as tissue-specific lipid modifications. The authors clustered alkylacyldiacylglycerols and deoxysphingolipids molecular species, reporting accumulation of deoxysphingolipids and ether-linked diacylglycerols in both tissues. Additional lipid subclasses were measured from Ando *et al.* (Additional information file 3), but those results were not discussed in the original publication ^10^. The results from Ando *et al.* are used here to show how BioPAN can provide a wider analysis of the lipid level changes and the interrelation between different lipid subclasses.

The datasets from the original paper, were downloaded as TXT files from Metabolomics Workbench (Project ID PR000713, Studies ID ST001065 and ST001066) and are available as underlying data (see *data availability* section). Each file was imported as described in the method implementation section. Pathway options and pathway calculations settings were as follows: Type: Lipid; Status: Active; Level: Lipid subclass; Subset of lipid data: Reactions; Condition of interest: Aged; Control condition: Young; *p* value: 0.05; Paired data: No.

**Figure 2.**
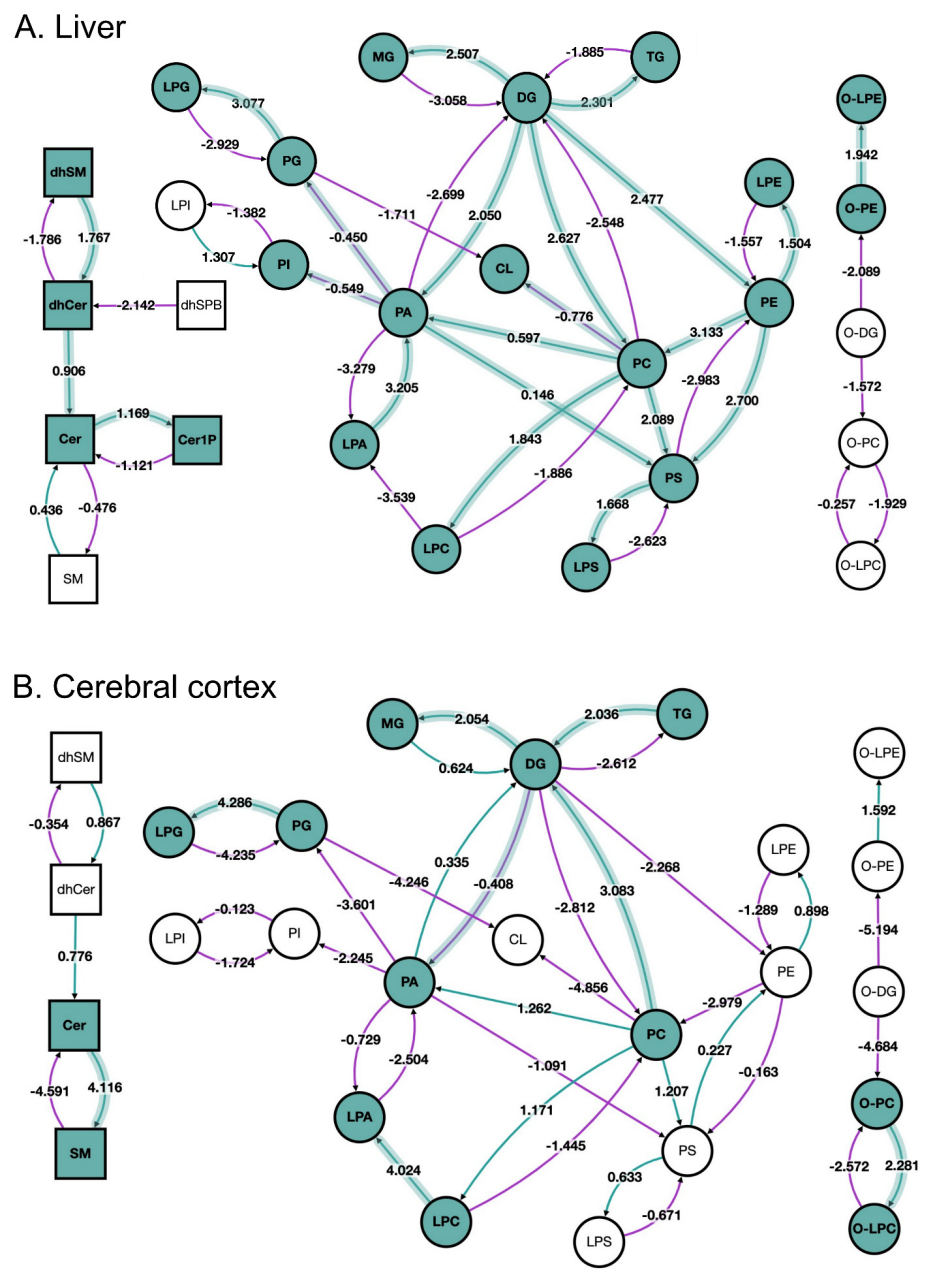
BioPAN lipid networks. Lipid network graphs exported from BioPAN for the liver ( **A**) and the cerebral cortex ( **B**) of aged mice compared to young mice ^17^. Green nodes correspond to active lipids and green shaded arrows to active pathways. Reactions with a positive Z score have green arrows while negative Z scores are coloured purple. Pathways options: aged condition of interest, young control condition, lipid type, active status, subclass level, reaction subset of lipid data, *p* value 0.05, and no paired-data.

The lipid network at the lipid subclass level is shown in Figure 2A for the liver and Figure 2B for the cerebral cortex. The lipid pathway graph allows the user to quickly spot trends and differences between the two sets of samples. Figure 2 shows that reactions using ether-linked diacylglycerol (O-DG) as a substrate are suppressed in both liver and cerebral cortex in old versus young, which agrees with the reported accumulation of this particular lipid subclass by Ando *et al*. ^17^. Here, BioPAN also found tissue-specific differences in lipid metabolism. For example, the reaction pathway results of aged mouse liver suggest active metabolism leading to accumulation of triacylglycerols (TG, Z-score = 2.301), a form of energy storage, contrary to a catabolic metabolism of TG observed in the cerebral cortex (Z-score = -2.612).

BioPAN also shows more differences in the metabolism of sphingolipids between the tissues. Specifically, in the liver the pathway is shifted towards the formation of ceramide-1-phosphate (Cer1P), while in the cerebral cortex sphingolipid metabolism points towards the formation of sphingomyelin (SM). Moreover, in the liver, glycerophospholipids and lysophospholipids synthesis is active with exception of LPA, contrary to what it is observed in the cerebral cortex, where glycerophospholipids and lysophospholipids pathways are suppressed, but production of LPA is favoured.

BioPAN provides tables showing the suggested genes known to be involved in each reaction. Table 1 shows the lipid subclass active reaction results generated for the liver dataset. For example, the pathway PE→PC→PS→LPS designates the genes *PEMT, PTDSS1, PLA2G2E, PLA2G2A, PLA2G2F*. It should be noted that the default nomenclature of the genes in BioPAN corresponds to human species. In the example case presented here, the lipidomics data was obtained from mice, and although most often human and mouse genes are the same, there can be subtle differences between species. Thus, the user is encouraged to assign the gene according to the species in their own study. To aid the user in identifying the species each gene in Table 1 has a link that exists in the LIPID MAPS ^®^ Proteome Database (LMPD) ( https://lipidmaps.org/resources/databases/index.php?tab=lmpd). For example, in Table 1 *PEMT* gene refers to a table containing the name and species of the gene, including a unique LMP ID for each gene/species pair. Thus, LMP002477 is assigned to the gene *Pemt* for mouse, which converts PE to PC. The status of this pathway might be changed by the loss of function or altered activity in any of the enzymes involved. Therefore, the list provided by BioPAN might help guide integration of lipidomics with proteomics or transcriptomics data as it directly suggests target proteins for further analysis.

**Table 1.** BioPAN predicted genes of the lipid active reactions in liver of aged vs young mice. Table contains the active reactions chains found by BioPAN according to the Z-score values. Several genes are predicted as being involved in the current status of each reaction based on their function. Pathway options: aged condition of interest, young control condition, lipid type, active status, subclass level, reaction subset of lipid data, *p* value 0.05, no paired-data.

lipid subclass active reaction (Aged vs Young)		
Reaction chains	Z-score	Predicted genes
PE→PC→PS→LPS	3.978	*PEMT, PTDSS1, PLA2G2E, PLA2G2A, PLA2G2F*
DG→PC→PS→LPS	3.686	*CHPT1, PTDSS1, PLA2G2E, PLA2G2A, PLA2G2F*
PE→PC→LPC	3.519	*PEMT, PLA2G2E, PLA2G2A, PLA2G2D, PLA2G2F, PLA2G1B*
LPA→PA→PG→LPG	3.367	*AGPAT1, AGPAT2, AGPAT3, AGPAT4, AGPAT5, CDS1, CDS2, PTPMT1, PLA2G4D, PLA2G4F, CRLS1*
PE→PC→PA→PG→LPG	3.179	*PEMT, PLD1, PLD2, CDS1, CDS2, PTPMT1, PLA2G4D, PLA2G4F, CRLS1*
DG→PC→LPC	3.161	*CHPT1, PLA2G2E, PLA2G2A, PLA2G2D, PLA2G2F, PLA2G1B*
PG→LPG	3.077	*PLA2G4D, PLA2G4F, CRLS1*
LPA→PA→PS→LPS	2.898	*AGPAT1, AGPAT2, AGPAT3, AGPAT4, AGPAT5, CDS1, PTDSS1, PLA2G2E, PLA2G2A, PLA2G2F*
DG→PE→LPE	2.815	*CEPT1, PLA2G4C*
PE→PS	2.700	*PTDSS2*
PC→PS→LPS	2.657	*PTDSS1, PLA2G2E, PLA2G2A, PLA2G2F*
DG→MG	2.507	*PNPLA2, PNPLA3*
DG→TG	2.301	*DGAT2*
DG→PC→PA	2.280	*CHPT1, PLD1, PLD2*
dhSM→dhCer→Cer→Cer1P	2.218	*SGMS1, SGMS2, DEGS1, DEGS2, CERK*
DG→PA	2.050	*DGKA, DGKB, DGKD, DGKE, DGKG, DGKH, DGKI, DGKK, DGKQ, DGKZ*
O-PE→O-LPE	1.942	*PLA2G4A*
LPA→PA→PI	1.878	*AGPAT1, AGPAT2, AGPAT3, AGPAT4, AGPAT5, CDS1, CDS2, CDIPT*
PC→LPC	1.843	*PLA2G2E, PLA2G2A, PLA2G2D, PLA2G2F, PLA2G1B*
PE→PC→PA→PI	1.837	*PEMT, PLD1, PLD2, CDS1, CDS2, CDIPT*
PS→LPS	1.668	*PLA2G2E, PLA2G2A, PLA2G2F*
PE→PC→CL	1.667	*PEMT, TAZ*

**Figure 3.**
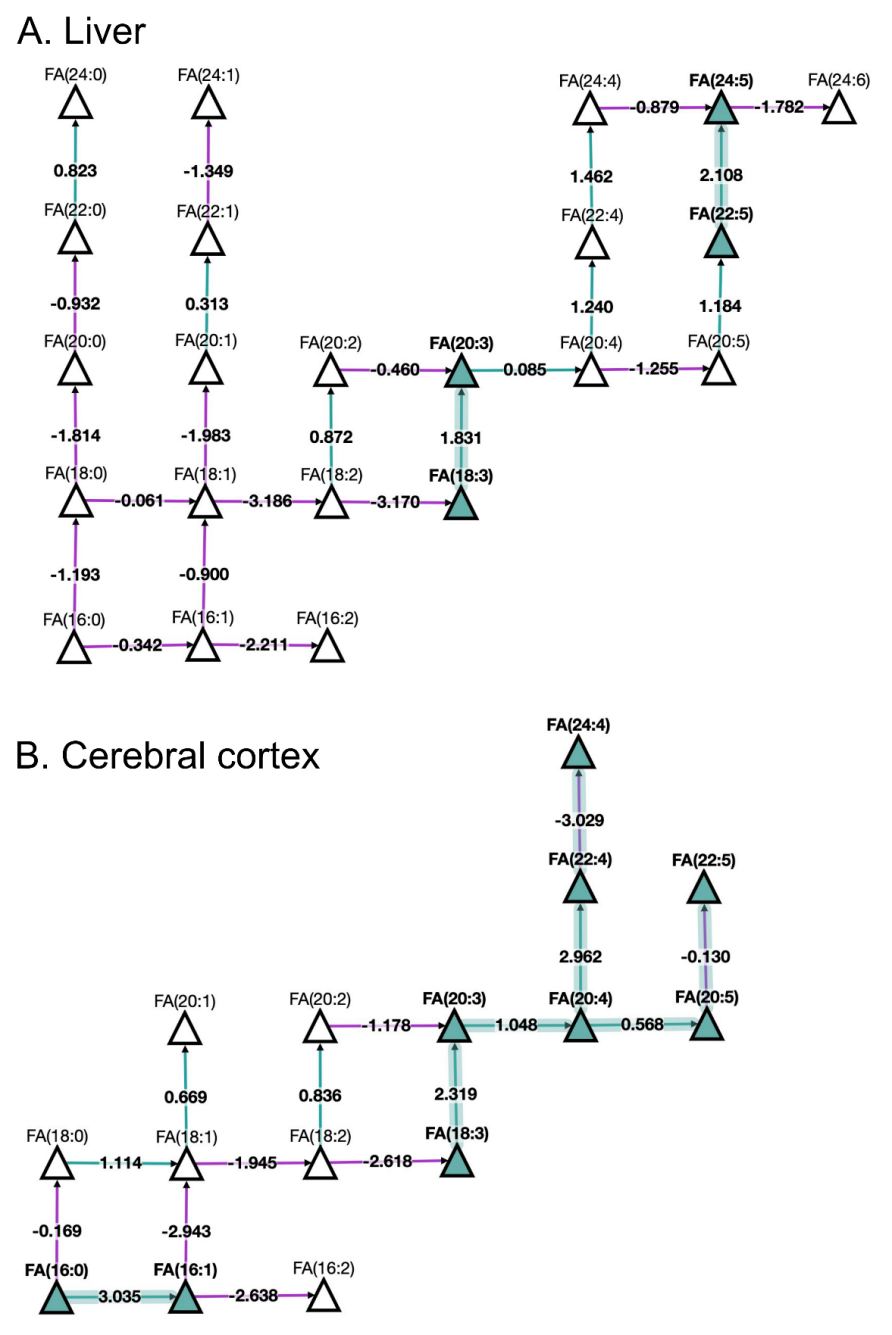
BioPAN fatty acids networks. FA graphs exported from BioPAN tool for the liver ( **A**) and the cerebral cortex ( **B**) of aged mice compared to young mice ^17^. Green nodes correspond to active lipids and green shaded arrows to active pathways. Reactions with a positive Z score have green arrows while negative Z scores are coloured purple. Pathways options: aged condition of interest, young control condition, lipid type, active status, subclass level, reaction subset of lipid data, *p* value 0.05, and no paired-data.

Looking at fatty acids (FA) pathways at the molecular species level with BioPAN ( Figure 3) shows the FA network obtained for liver and cerebral cortex of aged in comparison to young mice. The results show active metabolism towards the production of monounsaturated FA 16:1 and polyunsaturated FA 20:4, FA 20:5, and FA 22:4 in the cerebral cortex compared to the liver. Additionally, BioPAN has highlighted accumulation of the long acyl chain, polyunsaturated FA 24:5 in liver, which is associated with the activation of the *ELOVL2* gene found in Table 2. Moreover, the results shown in Table 2 suggest a general activation of elongases (ELOVL) rather than desaturases (SCD) which was found to be a common trait in both tissues.

**Table 2.** BioPAN predicted genes for fatty acid active reactions in liver of aged vs young mice. Table contains the active reaction chains found by BioPAN according to the Z-score values. Several genes are predicted as being involved in the current status of each reaction based on their function. Pathway options: aged condition of interest, young control condition, fatty acid type, active status, *p* value 0.05, no paired-data.

fa active reaction (Aged vs Young)		
Reaction chains	Z-score	Predicted genes
FA(22:5)→FA(24:5)	2.108	*ELOVL2*
FA(18:3)→FA(20:3)	1.831	*ELOVL5, ELOVL7*

## Conclusion

Modern lipidomics techniques generate a huge amount of data and information on many lipid subclasses, making the interpretation of results challenging. BioPAN provides several advantages over simpler analyses of individual lipids. The mapping of the lipid network graph provides a bigger picture allowing a new perspective on the data where new connections can be made, and a study can be moved forward into particular areas of interest. BioPAN offers a quick visualisation of differences in lipid pathways in mammal studies as well suggesting genes and their enzyme products involved in those differences.

## Data availability

### Underlying data

The datasets analysed as an example are available at the NIH Common Fund's National Metabolomics Data Repository (NMDR) website, the Metabolomics Workbench, https://www.metabolomicsworkbench.org/ where it has been assigned Project ID PR000713 (studies ST001065 and ST001066).

The data can be accessed directly via its Project DOI: https://dx.doi.org/10.21228/M8HM48, https://www.metabolomicsworkbench.org/data/DRCCMetadata.php?Mode=SetupDownloadResults&StudyID=ST001065 and https://www.metabolomicsworkbench.org/data/DRCCMetadata.php?Mode=SetupDownloadResults&StudyID=ST001066.

Open Science Framework: BioPAN: a web-based tool to explore mammalian lipidome metabolic pathways on LIPID MAPS, https://doi.org/10.17605/OSF.IO/PKD6B ^18^.

This project contains the modified dataset from Ando *et al*. ^17^ according to BioPAN’s requirements:

- Cerebral cortex dataset: https://osf.io/reavk/
- Liver dataset: https://osf.io/zd4as/

### Extended data

Open Science Framework: BioPAN: a web-based tool to explore mammalian lipidome metabolic pathways on LIPID MAPS, https://doi.org/10.17605/OSF.IO/PKD6B ^18^.

 This project contains the following extended data:

- Table S1, reaction table.
- Section S2, filtering and tree checkbox discussion.
- Figure S1, Filter tool example for acyl chains 38:4 and 38:5.

## Software availability

Software, documentation and tutorial video are available from: https://lipidmaps.org/biopan/.

Archived source code at the time of the publication: https://doi.org/10.17605/OSF.IO/PKD6B ^18^.

The user can try BioPAN locally by following the readme instruction file located in: https://osf.io/7g2dx/.

License: GNU GPL 3.0
