# Supplementary material for: BioPAN: a web-based tool to explore mammalian lipidome metabolic pathways on LIPID MAPS
Source: F1000Res. 2021 Jun 9;10:4. Originally published 2021 Jan 6. [Version 2] doi: 10.12688/f1000research.28022.2 (PMC7848852; doi:10.12688/f1000research.28022.2)
Supplement: Peer review history

# Peer review: Reviewer response for version 1

**DOI:** 10.5256/f1000research.30993.r76705

**Published:** 2021-03-09

**Authors:** Ginger Milne

**Affiliations:** 1 Department of Medicine, Vanderbilt University Medical Center, Nashville, TN, USA

The submitted manuscript explains a web-based tool on the Lipid Maps website that helps the user to follow biosynthetic and metabolic pathways of endogenous lipids (the lipidome) following mass spectrometric analysis. This program will help the user link lipidomics data with underlying endogenous processes. The authors have taken care to cite appropriate references and give examples of how to use this tool. Further, the tool itself has a link to an excellent web-based tutorial.

 Recommendation:

1. The introduction ends abruptly.  It reads as if there is a paragraph missing emphasizing the importance and expected use of this tool.

Are the conclusions about the tool and its performance adequately supported by the findings presented in the article?

Yes

Is the rationale for developing the new software tool clearly explained?

Partly

Is the description of the software tool technically sound?

Yes

Are sufficient details of the code, methods and analysis (if applicable) provided to allow replication of the software development and its use by others?

Yes

Is sufficient information provided to allow interpretation of the expected output datasets and any results generated using the tool?

Yes

Reviewer Expertise:

Lipidomics, mass spectrometry, oxidized lipid mediators, lipid metabolism.

I confirm that I have read this submission and believe that I have an appropriate level of expertise to confirm that it is of an acceptable scientific standard.

# Response

**Published:** 2021-05-19

**Authors:** Andrea F. Lopez-Clavijo

**Affiliations:** Babraham Institute, UK

We thank you the reviewer Ginger Milne for her comments.

 Recommendation:

- The introduction ends abruptly.  It reads as if there is a paragraph missing emphasizing the importance and expected use of this tool.

--> A sentence was added: “So, BioPAN aids for automatic integration of lipid metabolism with lipid profiles, finding strong relationships between lipid substrates and lipid products catalysed by active or suppressed enzymes.”

# Peer review: Reviewer response for version 1

**DOI:** 10.5256/f1000research.30993.r76703

**Published:** 2021-01-29

**Authors:** Phillip Whitfield

**Affiliations:** 1 Glasgow Polyomics and Institute of Infection, Immunity and Inflammation, College of Medical, Veterinary and Life Sciences, University of Glasgow, Glasgow, UK

This manuscript by Gaud *et al. *is focused on BioPAN, a software tool designed for the mapping of lipid networks in mammalian systems. In order to demonstrate the utility of the software an exemplar analysis is outlined using publicly available lipidomic data sets acquired from the cerebral cortex and liver of young and aged mice. This is an interesting article and the BioPAN software will be useful in visualisation outputs from lipidomic experiments. There are a few minor suggestions:

 Introduction: A sentence could be added to the end of the introductory text summarising the overall objective of the presented work.

 Methods: It would be useful to clarify whether p<0.05 is always equivalent to Z-score>1.645.

 Conclusion: The authors may wish to indicate how the software could be further developed.

Are the conclusions about the tool and its performance adequately supported by the findings presented in the article?

Yes

Is the rationale for developing the new software tool clearly explained?

Partly

Is the description of the software tool technically sound?

Yes

Are sufficient details of the code, methods and analysis (if applicable) provided to allow replication of the software development and its use by others?

Yes

Is sufficient information provided to allow interpretation of the expected output datasets and any results generated using the tool?

Yes

Reviewer Expertise:

Lipidomics.

I confirm that I have read this submission and believe that I have an appropriate level of expertise to confirm that it is of an acceptable scientific standard.

# Response

**Published:** 2021-05-19

**Authors:** Andrea F. Lopez-Clavijo

**Affiliations:** Babraham Institute, UK

We thank you reviewer Phillip Whitfield for his comments.

*There are a few minor suggestions*:

- *Introduction: A sentence could be added to the end of the introductory text summarising the overall objective of the presented work.*

--> A sentence was added: “So, BioPAN aids for automatic integration of lipid metabolism with lipid profiles, finding strong relationships between lipid substrates and lipid products catalysed by active or suppressed enzymes.”

- *Methods: It would be useful to clarify whether p<0.05 is always equivalent to Z-score>1.645.*

--> It was added: “… ***corresponding to*** *Z > 1.645).”*

- *Conclusion: The authors may wish to indicate how the software could be further developed.*

--> A sentence was added: “Further work into the BioPAN database will be carried out to incorporate new biosynthetic pathways, such as: cholesterol, eicosanoids, and oxidised lipid subclasses, among others. Addition of glucosyl and galactosyl ceramides (Glc/Gal-Cer) to complement to the existing sphingolipids pathway in BioPAN will also be considered.”

# Peer review: Reviewer response for version 1

**DOI:** 10.5256/f1000research.30993.r76702

**Published:** 2021-01-26

**Authors:** Amaury Cazenave Gassiot

**Affiliations:** 1 Department of Biochemistry, Yong Loo Lin School of Medicine, Singapore, Singapore

The article entitled “BioPAN: a web-based tool to explore mammalian lipidome metabolic pathways on LIPID MAPS” described an online tool for visualisation of biological pathways from quantitative lipidomics data. The tool is an important contribution to lipidomics data analysis pipeline that could help make sense of ever more complex dataset.

 General comments

- Well-written manuscript and an interesting contribution for those studying quantitative lipidomics datasets.
- Recommendation is to accept with minor revision and, if possible, implementation of a few suggestions.

Suggestions

1. With regards to the statistical model used by the software. Could the authors elaborate on whether correction for multiple testing is done for p-value calculations? In the “Pathway calculation” section, would it be possible to let the user input any value for the significance threshold, rather than choosing from a few drop-down options?
2. The “Filter” field in the “Pathway options” section is very useful; however, it seems to take only one value at a time. Would it be possible to include logical operators? For instance, one could filter for “PC 32:1 AND PE 32:1”, or “38:5 OR 38:6”.
3. Ultimately, the tool relies on information contained in the pathway database. While using an in-house dataset for testing the tool, a large number of species were unfortunately “unprocessed”. Could the authors suggest how community users could help enriching the database?
4. The export function is useful, but only allows for exports of .png and .jpg graphics, the resolution of which seems to depend on the zoom status in the display window. Would it be possible to add an option to export as vector graphics, .svg for example?

Minor comments

1. Page 1, Abstract, line 3: change “increase” to “increases”.
2. Page 2, Authors roles, line 1: change “visualization” to “visualisation”.
3. Page 5, right column, 3 ^rd^ paragraph, line 3-4: change “visualized” to “visualised”.
4. Page 9, Acknowledgements: change “In memorium” to “In memoriam”.

Are the conclusions about the tool and its performance adequately supported by the findings presented in the article?

Yes

Is the rationale for developing the new software tool clearly explained?

Yes

Is the description of the software tool technically sound?

Yes

Are sufficient details of the code, methods and analysis (if applicable) provided to allow replication of the software development and its use by others?

Yes

Is sufficient information provided to allow interpretation of the expected output datasets and any results generated using the tool?

Yes

Reviewer Expertise:

Lipidomics.

I confirm that I have read this submission and believe that I have an appropriate level of expertise to confirm that it is of an acceptable scientific standard.

# Response

**Published:** 2021-05-19

**Authors:** Andrea F. Lopez-Clavijo

**Affiliations:** Babraham Institute, UK

We thank you reviewer Amaury Cazenave Gassiot for his comments.

*Suggestions:*

- *With regards to the statistical model used by the software. Could the authors elaborate on whether correction for multiple testing is done for p-value calculations? *

--> BioPAN does not correct for multiple testing as it calculates a Z-score to find stronger relationships within the data, hence higher Z-scores represent a more interesting result than others.

- *In the “Pathway calculation” section, would it be possible to let the user input any value for the significance threshold, rather than choosing from a few drop-down options?*

--> The drop-down menu has been replaced to a text box and it has been incorporated into BioPAN. 

- *The “Filter” field in the “Pathway options” section is very useful; however, it seems to take only one value at a time. Would it be possible to include logical operators? For instance, one could filter for “PC 32:1 AND PE 32:1”, or “38:5 OR 38:6”.*

--> A paragraph was added in the Display options section: “The search box allows the user to search for one or two queries using the logical operators AND / OR. Selecting the AND operator, the user can view lipid subclasses or molecular species for which the name implies both queries. For example, searching for “PC” and “34:0” on the lipid molecular species graph displays lipids “PC(34:0)” and “O-PC(34:0)”. Choosing the OR operator allow the user to visualise lipids whose names includes one of the two searches. For instance, searching “LPC” or “LPA” on the subclasses graph displays lipids “LPC”, “O-LPC” and “LPA”.”.

- *Ultimately, the tool relies on information contained in the pathway database. While using an in-house dataset for testing the tool, a large number of species were unfortunately “unprocessed”. Could the authors suggest how community users could help enriching the database?*

--> A paragraph was added in the operation section to address the “unprocessed” molecular species within BioPAN: “The unprocessed category corresponds to lipid molecular species that did not have a matching molecular species, as a reactant and product, with the same number of carbons and double bonds. For example, a substrate molecular species, such as PA 34:2 should have a matching product with the same sum composition (e.g. DG 34:2).  Additionally, some biological reactions require fatty acid or fatty-acyl-CoA molecular species. So, a lack of these subclasses in the input file prevents BioPAN to build up the reaction between substrates and products. If the substrate or product is not part of other reaction within the pathway, BioPAN will report it as unprocessed, such is the case of triacylglycerides (TG) subclasses. On other instances, like phosphatidylcholine (PC) and lysophosphatidylcholine (LPC), where PC is the substrate in the reaction to produce phosphatidic acid (PA) and LPC is the substrate to produce lysophosphatidic acid (LPA), those lipid subclasses molecular species can be considered as processed by BioPAN.”.

- *The export function is useful, but only allows for exports of .png and .jpg graphics, the resolution of which seems to depend on the zoom status in the display window. Would it be possible to add an option to export as vector graphics, .svg for example?*

--> This capability of export graphics in SVG has been added and a sentence was included in the text “in several formats (JPEG / PNG / **SGV** / JSON / TXT)”.

 Minor comments

 1.         Page 1, Abstract, line 3: change “increase” to “increases”.

 Done, thank you

 2.         Page 2, Authors roles, line 1: change “visualization” to “visualisation”.

 Done, thank you

 3.         Page 5, right column, 3rdparagraph, line 3-4: change “visualized” to “visualised”.

 Done, thank you

 4.         Page 9, Acknowledgements: change “In memorium” to “In memoriam”.

 Done, thank you
